# Supplementary material for: CAR-T cell therapy targeting surface expression of TYRP1 to treat cutaneous and rare melanoma subtypes
Source: Nat Commun. 2024 Feb 9;15:1244. doi: 10.1038/s41467-024-45221-2 (PMC10858182; doi:10.1038/s41467-024-45221-2)
Supplement: Supplementary file 1 — Supplementary Information [file 41467_2024_45221_MOESM1_ESM.pdf]

Supplementary Figure 1

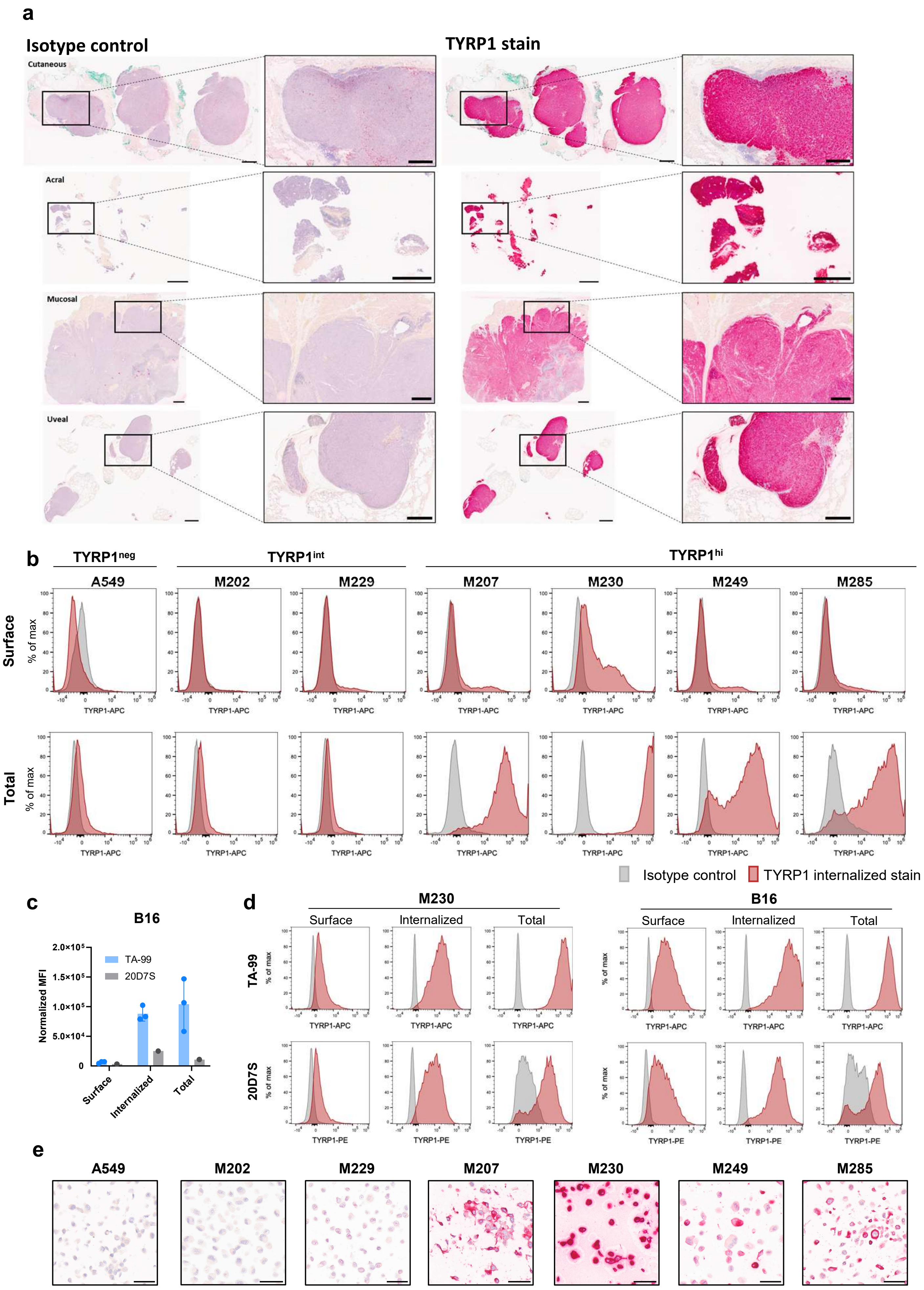

Supplementary Figure 1. A. TYRP1 expression in metastatic tumor lesion of cutaneous, acral, mucosal, and uveal melanoma. A. TYRP1 expression measured by immunohistochemistry stain. Scale bar equal to 2mm. Full tissue and magnified sections (scale bar equal to 1mm) are shown for added detail. Isotype control is shown on the left panels, and TYRP1 stain on the right panels. One representative image of cutaneous (n=11), acral (n=10), mucosal (n=6), and uveal (n=11) melanoma is shown. **B.** Surface (top) and total (bottom) expression of *TYRP1* of TYRP1 (red) compared to the isotype control (gray) in cell lines with high (≥7 Log2 FPKM), intermediate (≥1 Log2 FPKM) and negative (<1 Log2 FPKM) *TYRP1* RNA levels. One representative out of three replicates is shown. **C.** Surface, surface/internalized over a 16h period and total TYRP1 expression in B16 cells stained with two TYRP1 antibodies (TA-99 and 20D7SL) and normalized with the isotype control (mean ± SD, n=1 for 20D7S and n=3 for TA-99). **D.** Surface, surface/internalized over a 16h period and total expression of TYRP1 (red) compared to the isotype control (gray) in M230 (left) and B16 cells (right) stained with TA-99 (top) or 20D7S (bottom) antibodies. One representative out of three replicates is shown (TA-99), one datapoint is shown for 20D7S. **E**. TYRP1 expression in cell lines in vitro measured by immunohistochemistry stain, one representative area out of the slide is shown. Scale bar equal to 50um. Source data and exact p values are provided as a Source Data file.

Supplementary Figure 2

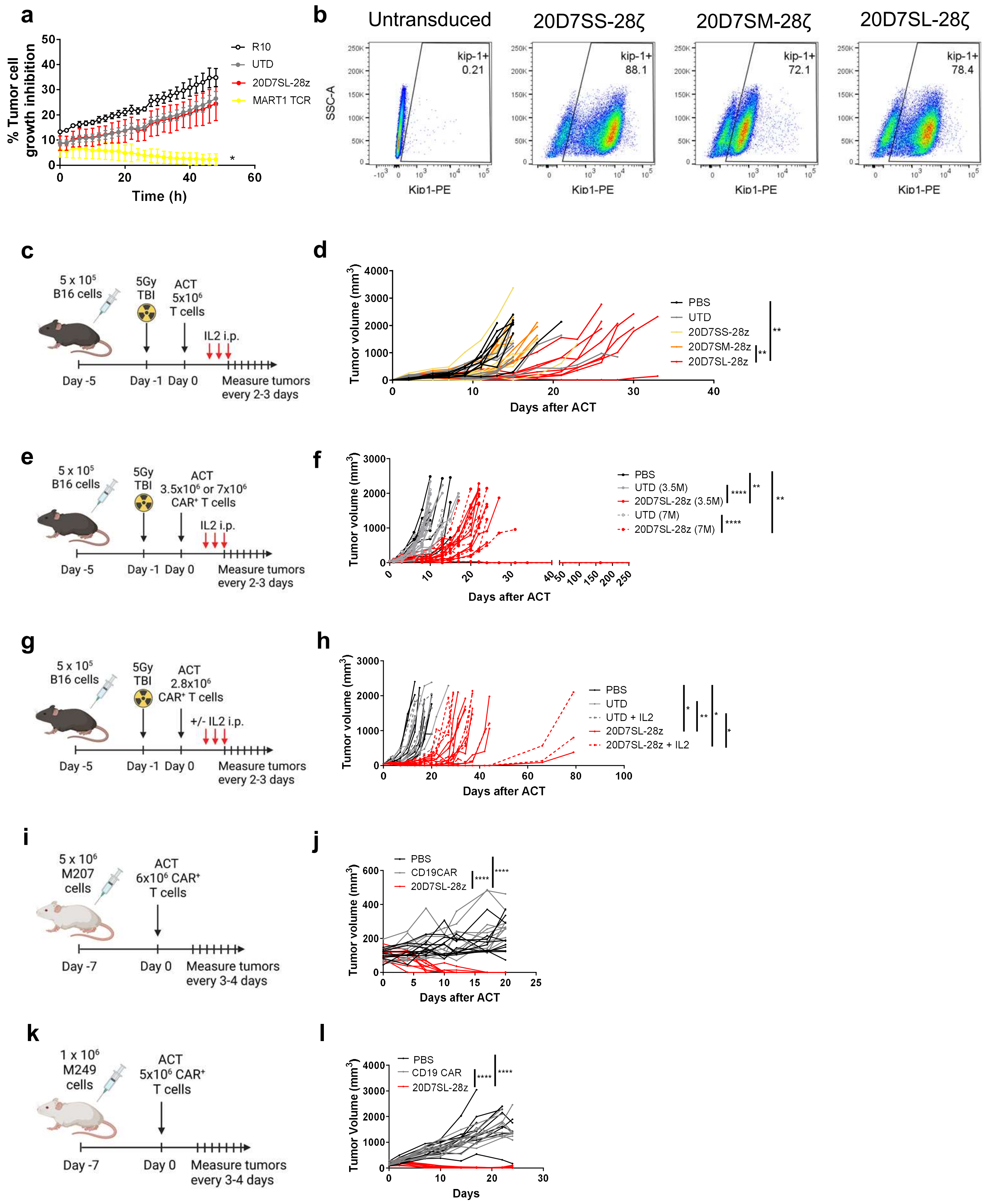

Supplementary Figure 2. TYRP1 CAR with a long flexible hinge exhibits superior tumor control in TYRP1^{high} syngeneic and patient-derived melanoma models. A. Antitumor activity of the MART-1 TCR and 20D7SL-28ζ CAR-T cells upon co-culture with M202 melanoma cells at 1:1 E:T ratios. Mean ± SD is plotted (n=3). *p<0.05 MART1 TCR vs untransduced cells, unpaired, two-tailed t test with Holm-Sidak adjustment for multiple comparisons. **B.** Murine primary T cells transduced with the TYRP1 CARs. Representative flow cytometry plots showing CAR expression on the cell surface. CAR expression was detected with the Kip-1 anti-whitlow linker antibody. **C, E, G, I, K.** Schematics of the *in vivo* mouse studies indicating the timeline, tumor cell and CAR-T cell doses, and irradiation and IL-2 doses and timelines if applicable. Graphical depictions were created with BioRender.com. **D, F, H, J, L.** Kinetic of tumor growth or regression over time after treatment with 20D7S-derived CAR-T cells alone or in combination with IL-2. Untransduced T cells or CD19 CAR-T cells and vehicle were used as controls. Tumor size was measured with a caliper. Single animal values are plotted (n=5-10, exact sample sizes provided in main figure 3).

* p<0.05, ** p<0.005, **** p<0.0005 unpaired t test with Holm-Sidak adjustment for multiple comparisons. Source data and exact p values are provided as a Source Data file.

Supplementary Figure 3

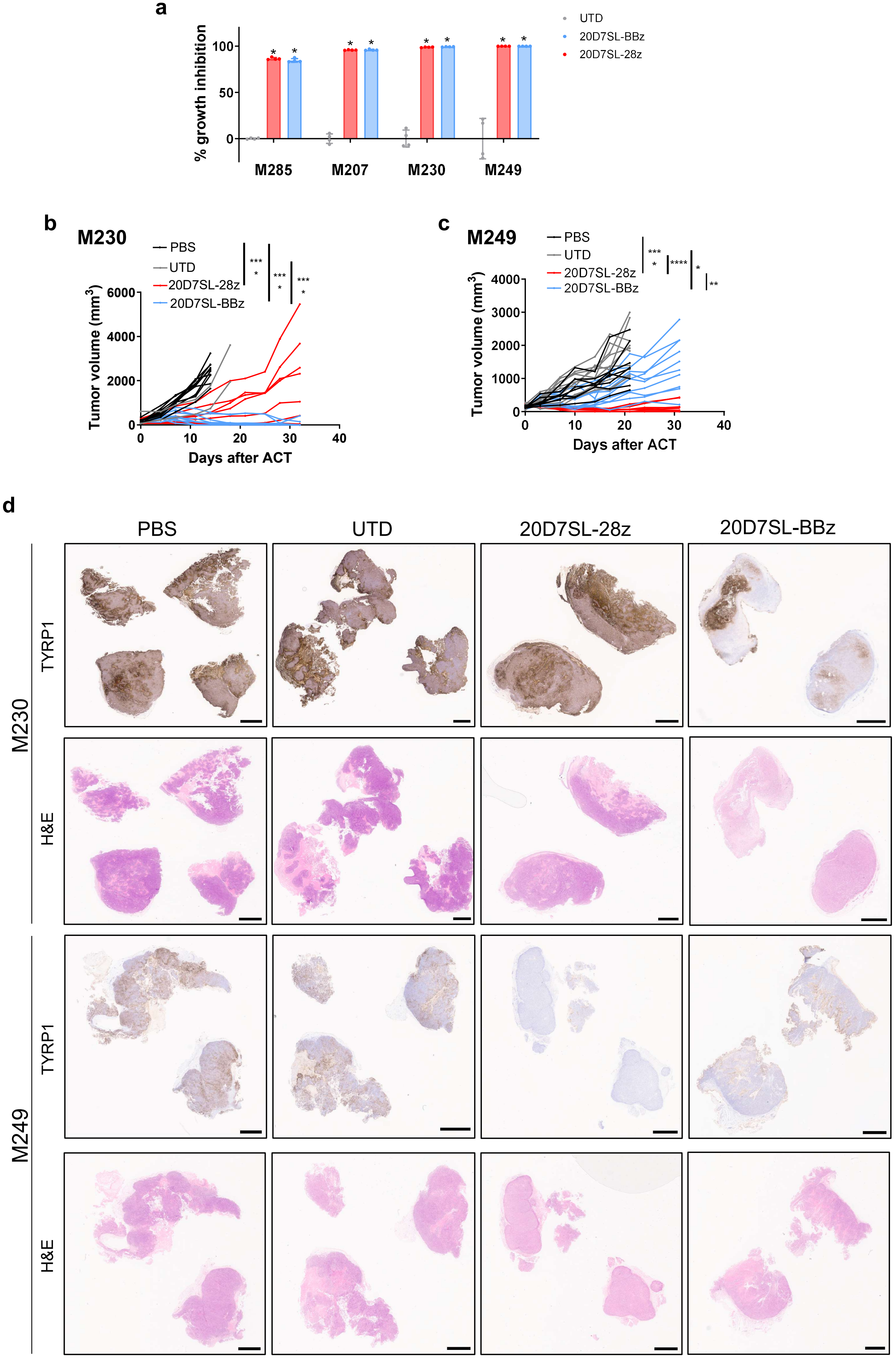

Supplementary Figure 3. CD28 outperforms 4-1BB as the costimulatory signal for the 20D7SL CAR, leading to antitumor activity in a larger tumor panel. A. Co-culture of 20D7SL- 28ζ and 20D7SL-BBζ CAR-T cells with a panel of $TYRP^{high}$ patient-derived melanoma cells. Cytotoxicity is shown as the percentage of tumor growth inhibition normalized by the growth of the cell lines co-cultured with untransduced T cells at 96h after co-culture at a 1:1 E:T ratio. Untransduced T cells (UTD) are used as a negative control. Mean ± SD are plotted (n=4). *p<0.05 vs untransduced cells, unpaired, two-tailed t test with Holm-Sidak adjustment for multiple comparisons. **B, C.** Kinetic of tumor growth or regression over time after treatment with 20D7SL- 28ζ and the 20D7SL-BBζ CAR-T cells in NSG mice bearing M230 **(B)** and M249 **(C)** subcutaneous tumors. Untransduced T cells and PBS were used as controls. Tumor size was measured with a caliper. Single animal data are plotted (n=8-10, exact sample sizes provided in main figure 4). * p<0.05, ** p<0.005, **** p<0.0005 unpaired t test with Holm-Sidak adjustment for multiple comparisons. **D**. TYRP1 expression measured by immunohistochemistry stain. Scale bar equal to 2mm. Each image shows all tumors from one representative mouse in each group. For each cell line, we show TYRP1 staining on top and H&E staining in the bottom. Source data and exact p values are provided as a Source Data file.

Supplementary Figure 4

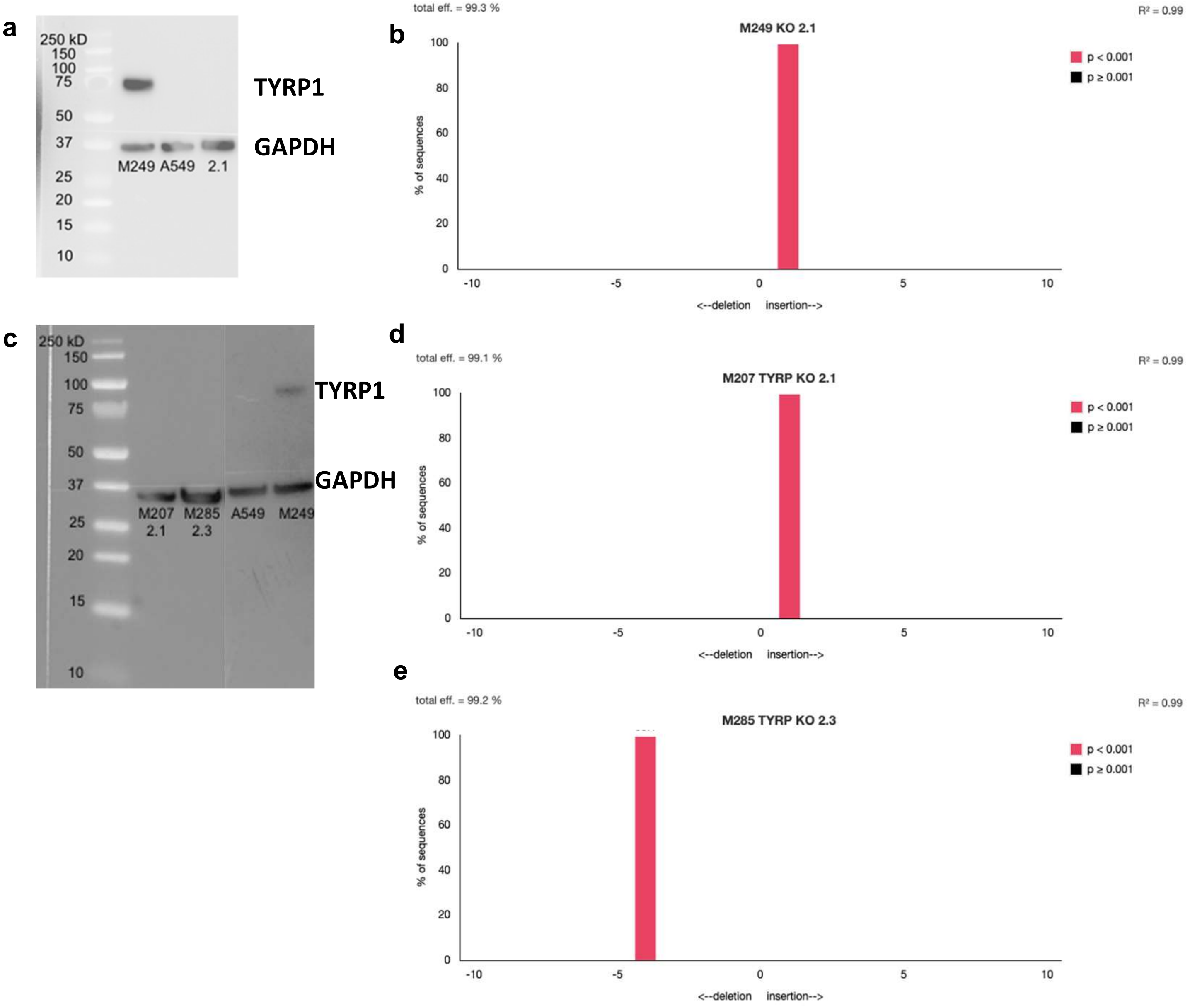

Supplementary Figure 4. Generation and validation of *TYRP1*-knockout cell lines. A, C. TYRP1 detection by Western blot in the M249 TYRP1 KO clone 2.1 **(A)**, M207 TYRP1 KO clone 2.1, and M285 TYRP1 KO clone 2.3 **(C)**. Protein extracts from parental A549 and M249 are used as negative and positive control, respectively. GAPDH detection is used as a loading control. Complete gel images are shown. One Western blot was run and is shown. **B, D, E.** TIDE analysis results showing pure knockout cell lines with 1 base pair insertion for M249 TYRP1 KO clone 2.1 **(B)**, 1 base pair insertion for M207 TYRP1 KO clone 2.1 **(D)**, and 4 base pair deletion in M285 TYRP1 KO clone 2.3 **(E)**. Source data and exact p values are provided as a Source Data file.

Supplementary Figure 5

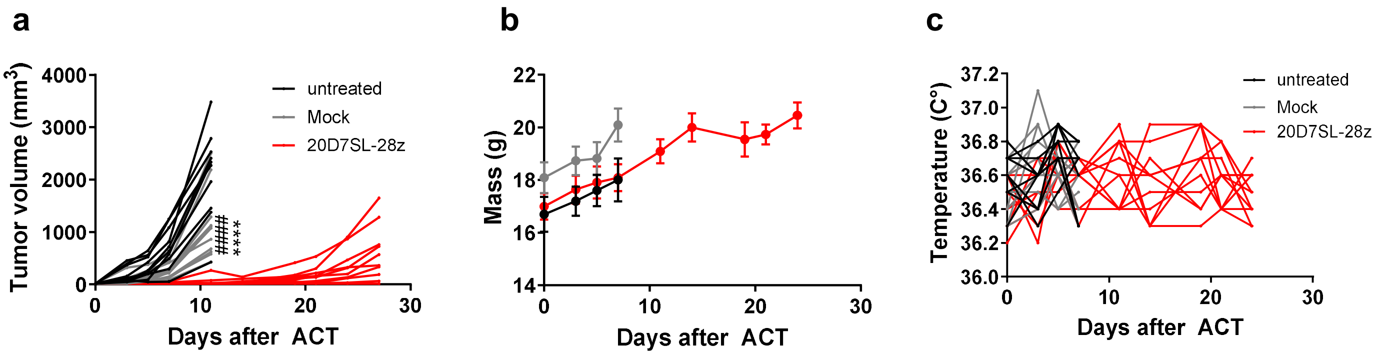

Supplementary Figure 5. 20D7SL-28ζ CAR-T cells exert strong tumor control and lack of body weight and whole-body temperature changes in the immunocompetent B16 melanoma model. A. Kinetic of tumor growth or regression over time after treatment with 20D7SL-28ζ CAR-T cells. Tumor size was measured with a caliper. Single animal values are plotted, exact sample sizes provided in main figure 6. **B.** Body weight over the course of the study. Mean ± SD are plotted. **C.** Whole-body temperature over the course of the study. Single animal values are plotted. Untransduced T cells and vehicle were used as controls. (for **B** and **C**, untreated n=10, UTD and 20D7SL-28z n=11). Source data and exact p values are provided as a Source Data file.

Supplementary Figure 6

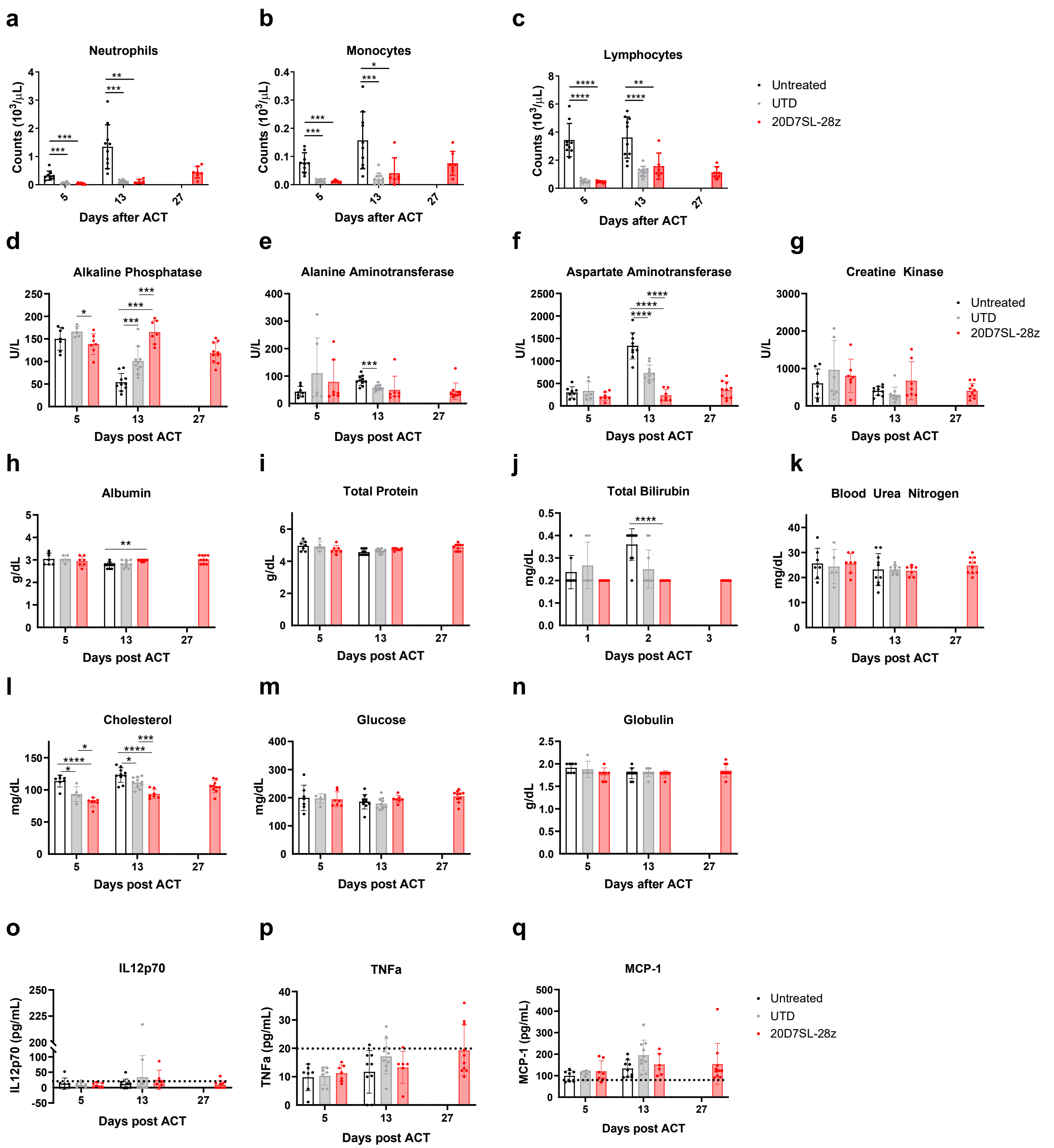

Supplementary Figure 6. Cell blood counts differential, serum chemistry and, cytokine release after treatment with 20D7SL-28ζ CAR-T cells in immunocompetent B16 melanoma model. A–C. Cell blood counts differential at days 5, 13, and 27 after adoptive T cell transfer with 20D7SL-28ζ CAR-T cells. Neutrophils **(A)**, Monocytes **(B)**, and Lymphocytes **(C)** are shown. Mean ± SD are plotted (n=6-10, exact sample sizes provided in main figure 6). **D-N.** Serum chemistry at days 5, 13, and 27 after adoptive T cell transfer with 20D7SL-28ζ CAR-T cells. Alkaline phosphatase **(D)**, alanine aminotransferase **(E)**, aspartate aminotransferase **(F)**, creatine kinase **(G)**, albumin **(H)**, total protein **(I)**, total bilirubin **(J)**, blood urea nitrogen **(K)**, cholesterol **(L)**, glucose **(M)**, globulin **(N)** are shown. Mean ± SD are plotted (n=5-10). **O-Q.** Cytokine release in serum at days 5, 13, and 27 after adoptive T cell transfer with 20D7SL-28ζ CAR-T cells. IL12p70 **(O)**, TNFα **(P)**, and MCP-1 **(Q)** are shown. Mean ± SD are plotted (n=6-10, exact sample sizes provided in main figure 6l-n). Black dashed line shows the minimum quantifiable levels using the BD CBA Mouse Inflammation Kit (20pg/mL for IL12p70 and TNFα, 80pg/mL for MCP-1). In all plots, untransduced T cells and vehicle were used as controls. *$p<0.05$, ** $p<0.005$, *** $p< 0.001$, **** $p<0.0005$ unpaired t test with Holm-Sidak adjustment for multiple comparisons. Unless otherwise indicated, differences are not statistically significant. Source data and exact p values are provided as a Source Data file.

Supplementary Figure 7

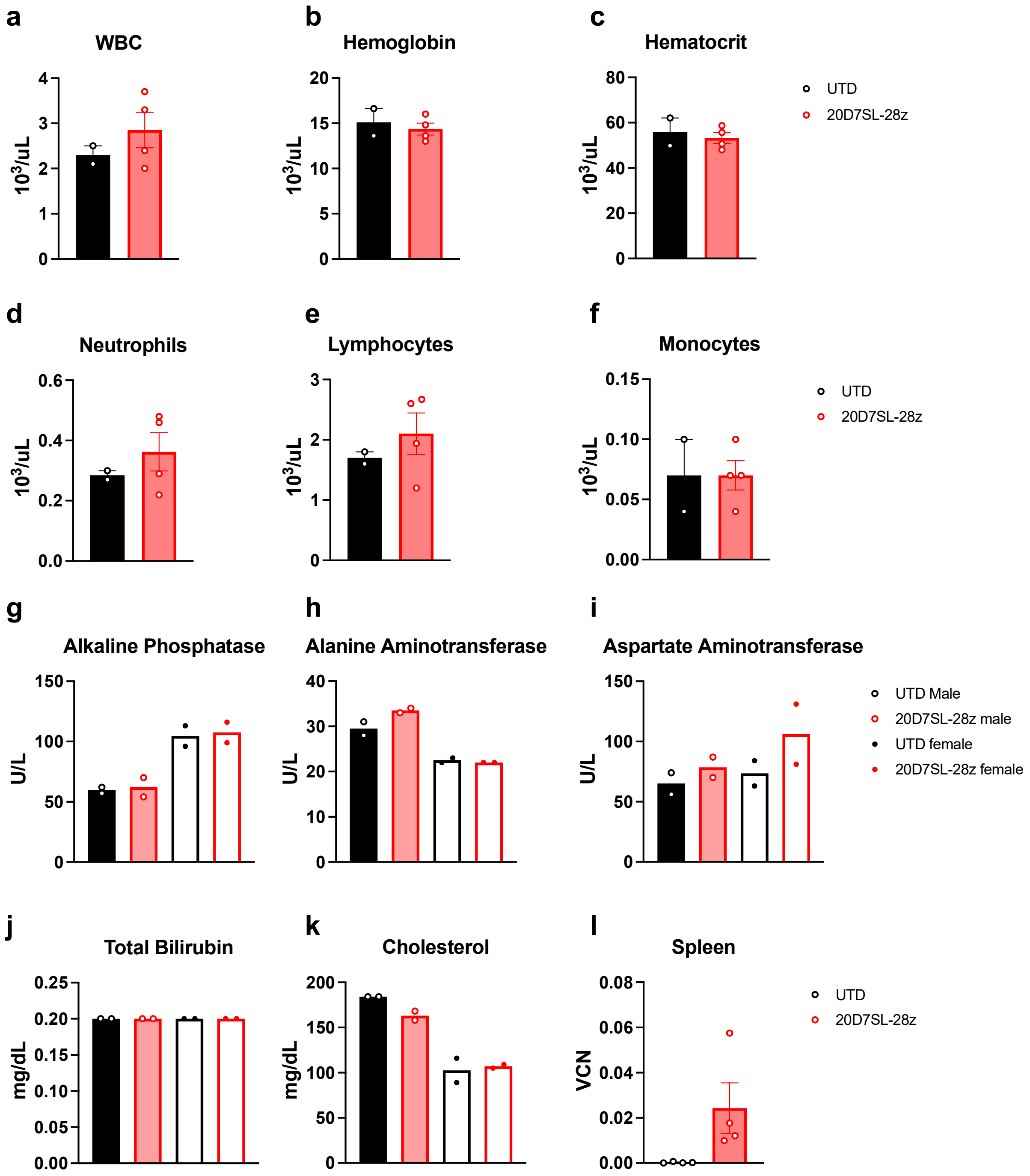

Supplementary Figure 7. Long-term toxicity assessment in the immunocompetent B16 melanoma model. A–F. Complete blood counts and differential at day 90 after adoptive T cell transfer with 20D7SL-28ζ CAR-T cells. White blood cells **(A)**, hemoglobin **(B)**, hematocrit **(C)**, neutrophils **(D)**, lymphocytes **(E),** and monocytes **(F)** are shown. Mean ± SD are plotted (n=4). Unpaired, two-sided t test (all data non-significant). **G–K.** Serum chemistry at day 90, after adoptive T cell transfer with 20D7SL-28ζ CAR-T cells. Alkaline phosphatase **(G)**, Alanine aminotransferase **(H)**, aspartate aminotransferase **(I)**, total bilirubin **(J),** and cholesterol **(K)** are shown. Mean ± SD are plotted (n=2). Unpaired, two-sided t test for males and females (no significant differences). **L.** Retrovirus vector copy number in the spleen of mice without tumors treated with 20D7SL-28ζ CAR-T cell therapy at day 90 after adoptive T cell transfer. Mean ± SD are plotted (n=4). Unpaired, two-sided t test (no significant differences). In all plots untransduced T cells were used as controls. Source data and exact p values are provided as a Source Data file.

Supplementary Figure 8

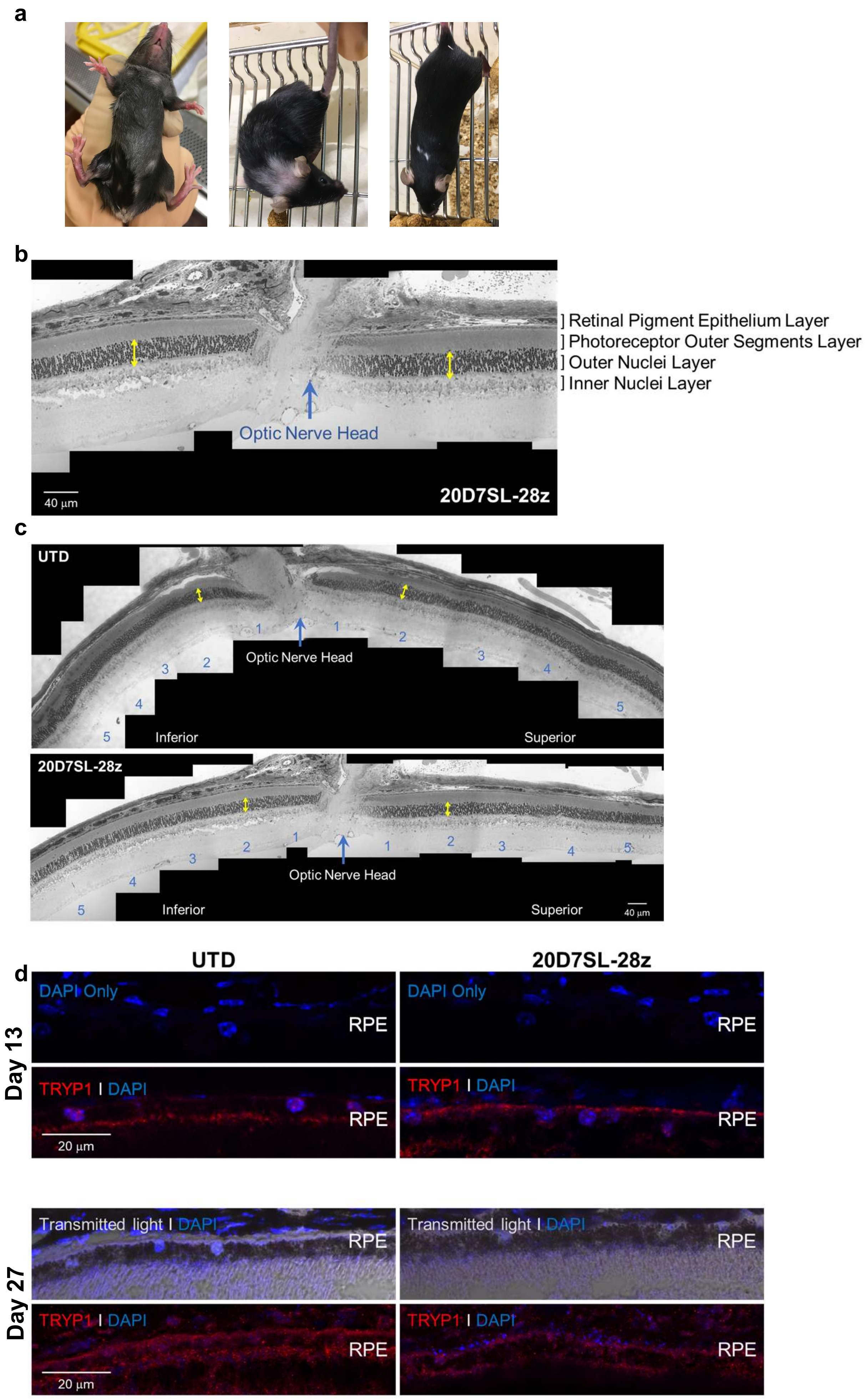

Supplementary Figure 8. On-target off-tumor toxicity in the skin and the retinal pigmented epithelium. A. Sporadic cases of vitiligo in immunocompetent mice with long-term complete responses to 20D7SL-28ζ CAR-T cell therapy. Images of three mice treated with 20D7SL-28ζ CAR-T cells that presented with vitiligo after long-term remission of their tumor lesions. **B-C.** Retinal morphology. Zoom-in representative image of retina collected at day 13 after ACT in n=4 mice receiving 20D7SL-28ζ CAR-T cells for identification of key retina layers and the optic nerve head evaluated by light microscopy: retinal pigment epithelium (RPE), photoreceptor outer segments layer, and outer nuclei layer (ONL). **C.** Reconstructed representative images of five frames superior and inferior to the optic nerve head of retinas collected at day 13 after ACT in mice receiving untransduced T cells (top) or 20D7SL-28ζ CAR-T cells (bottom). Both groups received total body irradiation. Yellow arrows indicate the thickness of the outer nuclei layers measurements taken at different frames. **D.** Representative confocal microscopy images of TYRP1 staining in the RPE from mice at days 13 and 27 after receiving ACT with untransduced T cells (left) or 20D7SL-28ζ CAR-T cells (right). One image from n=4 mice, three sections of the retina per mouse. Source data and exact p values are provided as a Source Data file.

Supplementary Figure 9

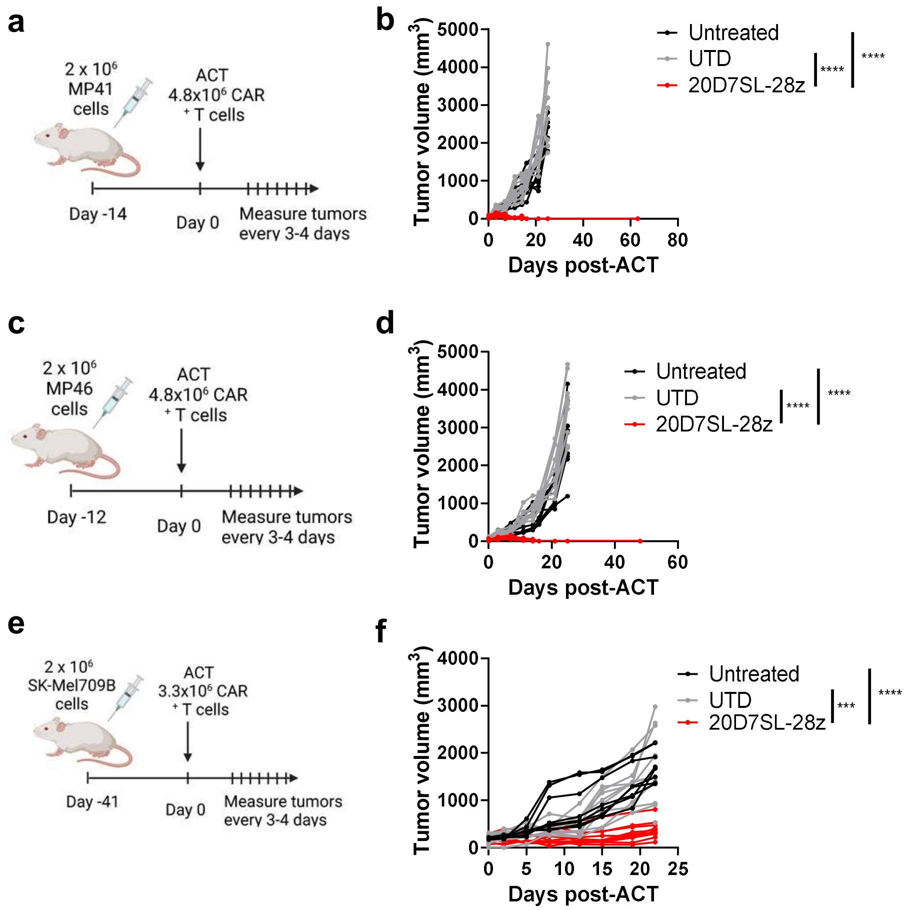

Supplementary Figure 9. 20D7SL-28ζ CAR-T cell therapy is effective at treating acral and uveal melanoma. A, C, E. Schematics of the *in vivo* mouse studies indicating the timeline and the tumor cell and CAR-T cell doses. Graphical depictions were created with BioRender.com. **B, D, F.** Antitumor activity of the 20D7SL-28ζ CAR-T cells *in vivo* in patient-derived models of uveal and acral melanoma in immunodeficient mice. Kinetic of tumor growth or regression over time after treatment with 20D7SL-28ζ CAR-T cells. Untransduced T cells and vehicle are used as controls. Single animal values are plotted (n=8-10, exact sample sizes provided in main figure 7). *** p< 0.001, **** p<0.0005 unpaired t test with Holm-Sidak adjustment for multiple comparisons. Source data and exact p values are provided as a Source Data file.

Supplementary Figure 10

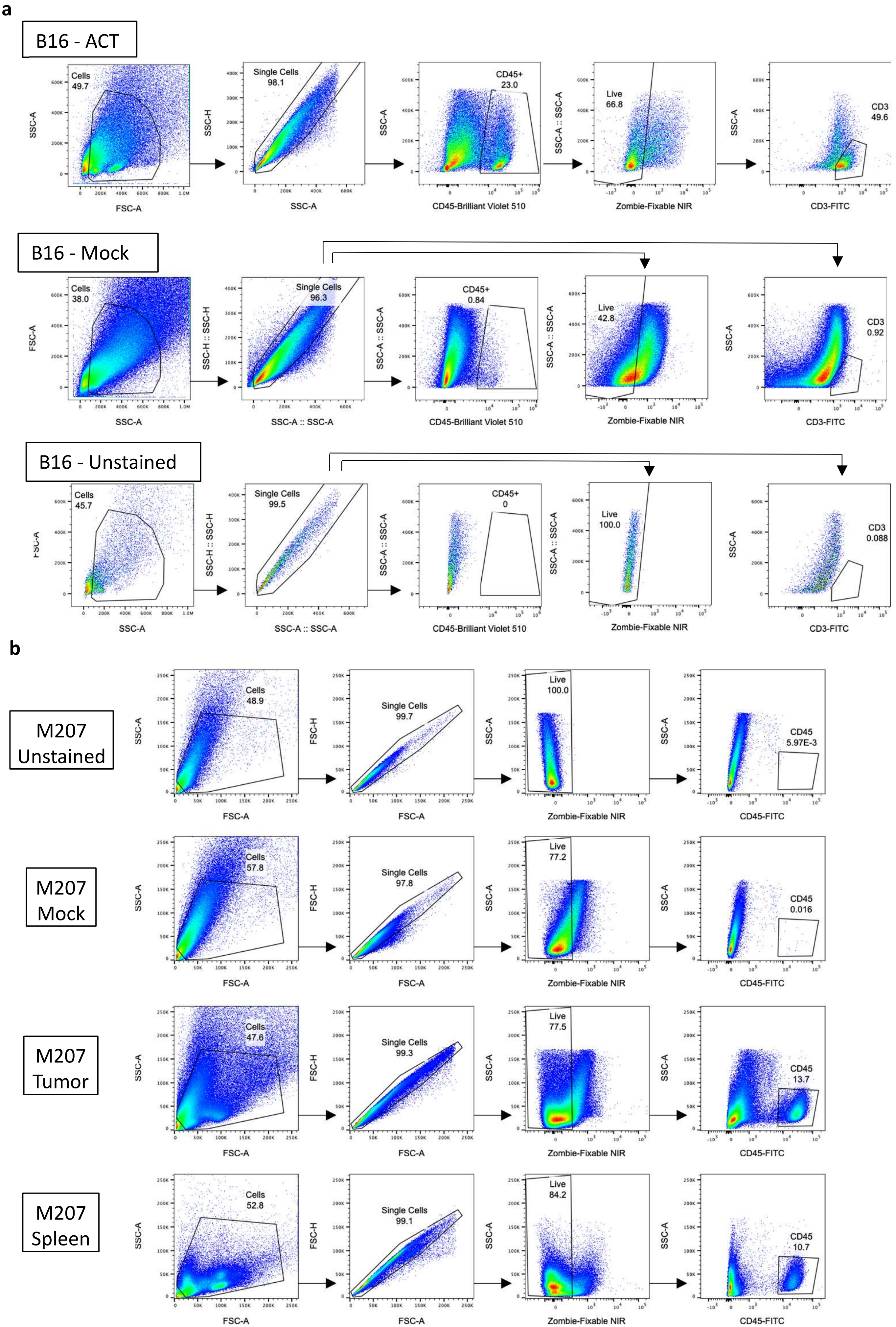

Supplementary Figure 10. Gating strategy for CAR-T cell detection in spleen and tumors from mice. A) Gating strategy followed to calculate the percentage of CD45+CD3+ cells (of single cells) in B16 tumors. A representative example of the unstained, a mock (untransduced T cells)- treated and a 20D7SL-treated (ACT) mouse are shown. B) Gating strategy followed to calculate the percentage of CD45^{+} T cells in tumors and spleens from M207 tumor-bearing NSG mice. One representative example of the unstained, a mock (untransduced T cells)-treated, a 20D7SL- treated (ACT) tumor and a spleen are shown. Source data and exact p values are provided as a Source Data file.

Supplementary Figure 11

Acral melanoma

Mock

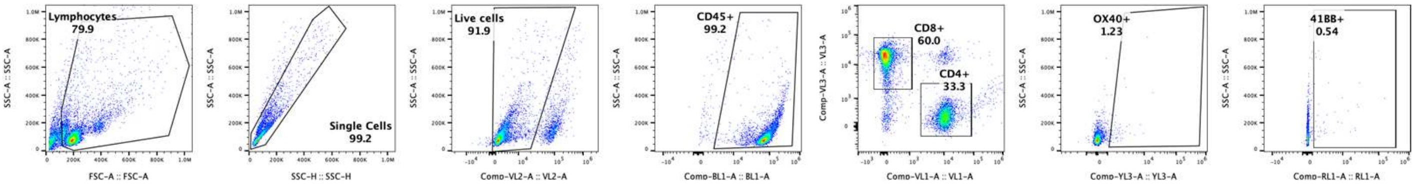

CAR

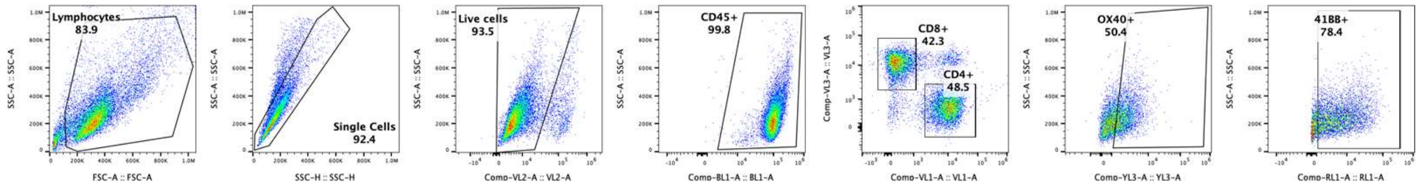

Uveal melanoma

Mock

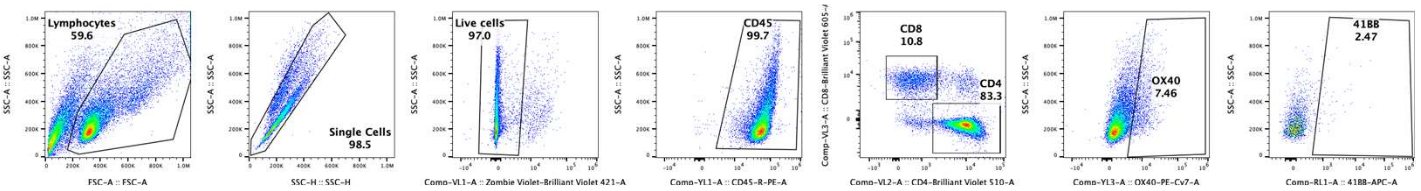

CAR

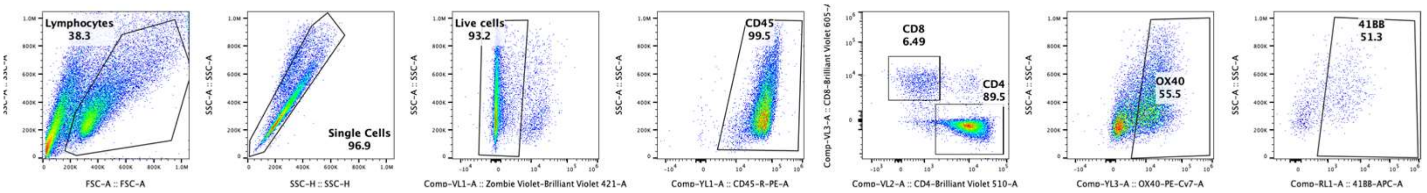

Supplementary Figure 11. Gating strategy for CAR-T cell activation markers measurement. Gating strategy followed to calculate the percentage of CD8+ T cells overexpressing 4-1BB and CD4+ T cells overexpressing OX-40. One representative untransduced T cell sample and CAR- T cell sample are shown. Source data and exact p values are provided as a Source Data file.
