# Supplementary material for: CAR-T cell therapy targeting surface expression of TYRP1 to treat cutaneous and rare melanoma subtypes
Source: Nat Commun. 2024 Feb 9;15:1244. doi: 10.1038/s41467-024-45221-2 (PMC10858182; doi:10.1038/s41467-024-45221-2)
Supplement: Supplementary file 3 — Reporting Summary [file 41467_2024_45221_MOESM3_ESM.pdf]

nature portfolio

Corresponding author(s): Cristina Puig Saus

Last updated by author(s): Dec 22, 2023

Reporting Summary

Nature Portfolio wishes to improve the reproducibility of the work that we publish. This form provides structure for consistency and transparency in reporting. For further information on Nature Portfolio policies, see our Editorial Policies and the Editorial Policy Checklist.

Statistics

For all statistical analyses, confirm that the following items are present in the figure legend, table legend, main text, or Methods section.

n/a	Confirmed	
[ ]	[x]	The exact sample size (*n*) for each experimental group/condition, given as a discrete number and unit of measurement
[ ]	[x]	A statement on whether measurements were taken from distinct samples or whether the same sample was measured repeatedly
[ ]	[x]	The statistical test(s) used AND whether they are one- or two-sided<br>*Only common tests should be described solely by name; describe more complex techniques in the Methods section.*
[x]	[ ]	A description of all covariates tested
[ ]	[x]	A description of any assumptions or corrections, such as tests of normality and adjustment for multiple comparisons
[ ]	[x]	A full description of the statistical parameters including central tendency (e.g. means) or other basic estimates (e.g. regression coefficient) AND variation (e.g. standard deviation) or associated estimates of uncertainty (e.g. confidence intervals)
[ ]	[x]	For null hypothesis testing, the test statistic (e.g. *F*, *t*, *r*) with confidence intervals, effect sizes, degrees of freedom and *P* value noted<br>*Give P values as exact values whenever suitable.*
[x]	[ ]	For Bayesian analysis, information on the choice of priors and Markov chain Monte Carlo settings
[x]	[ ]	For hierarchical and complex designs, identification of the appropriate level for tests and full reporting of outcomes
[x]	[ ]	Estimates of effect sizes (e.g. Cohen's *d*, Pearson's *r*), indicating how they were calculated

Our web collection on statistics for biologists contains articles on many of the points above.

Software and code

Policy information about availability of computer code

Data collection	Flow cytometry data was collected using Invitrogen Attune NxT flow cytometry software and analysed with FlowJo (V10.5.1)
Data analysis	ggplot2 package in R software (version 3.5.1), FlowJo (version 10.5.0) and GraphPad Prism (version 7.05).

For manuscripts utilizing custom algorithms or software that are central to the research but not yet described in published literature, software must be made available to editors and reviewers. We strongly encourage code deposition in a community repository (e.g. GitHub). See the Nature Portfolio guidelines for submitting code & software for further information.

Data

Policy information about availability of data

All manuscripts must include a data availability statement. This statement should provide the following information, where applicable:

- Accession codes, unique identifiers, or web links for publicly available datasets
- A description of any restrictions on data availability
- For clinical datasets or third party data, please ensure that the statement adheres to our policy

All sequencing data used had been previously published and can be bound in TCGA Research Network (http://cancergenome.nih.gov/), the Abril-Rodriguez cohort (doi:10.1038/s43018-019-0003-0), the Grasso cohort (doi:10.1016/j.ccell.2020.08.005), and the Ribas Laboratory melanoma cell line panel transcriptomic dataset published in (doi:10.1016/j.ccell.2018.03.017).

Research involving human participants, their data, or biological material

Policy information about studies with human participants or human data. See also policy information about sex, gender (identity/presentation), and sexual orientation and race, ethnicity and racism.

Reporting on sex and gender	Sex and gender were not considered in the study design.
Reporting on race, ethnicity, or other socially relevant groupings	No socially constructed or socially relevant categorization variables were used in this manuscript.
Population characteristics	Relevant population characteristics are described in Abril-Rodriguez et al. (doi:10.1038/s43018-019-0003-0) and Grasso et al. (doi:10.1016/j.ccell.2020.08.005). These population characteristics were not relevant for this manuscript.
Recruitment	Abril-Rodriguez et al. (doi:10.1038/s43018-019-0003-0) and Grasso et al.(doi:10.1016/j.ccell.2020.08.005) were randomized clinical trials.
Ethics oversight	No Study Protocol was used in this manuscript.

Note that full information on the approval of the study protocol must also be provided in the manuscript.

Field-specific reporting

Please select the one below that is the best fit for your research. If you are not sure, read the appropriate sections before making your selection.

[x] Life sciences   [ ] Behavioural & social sciences   [ ] Ecological, evolutionary & environmental sciences

For a reference copy of the document with all sections, see nature.com/documents/nr-reporting-summary-flat.pdf

Life sciences study design

All studies must disclose on these points even when the disclosure is negative.

Sample size	For in vitro experiments a minimum of n=3 was used to be able to perform statistical tests. For in vivo tumor growth experiments, a minimum of n=8 tumors were measured to obtain meaningful data with the least amount of animals. For other in vivo data point measurements a minimum of n=4 samples were tested to allow for statistical analysis.<br>No statistical method was performed to determine sample size. These n is a standard sample size for these experiments and was sufficient to assess the CAR reactivity, antitumor activity and activation.
Data exclusions	No data has been excluded from the analysis.
Replication	Biological replicates have been used throughout the study to verify the reproducibility of experimental findings. For in vitro T cell functional studies 3 biological replicates per condition were performed in each experiment unless otherwise indicated. This n is a standard sample size for these experiments and was sufficient to evaluate T cell functionality. All replicates were successful.
Randomization	For in vivo therapeutic efficacy studies, animals were divided into experimental groups according to their tumor size, aiming to have similar tumor sizes in all groups.<br>For toxicity studies, randomization was done on the day of irradiation and mice were allocated into treatment groups without any specific criteria.<br>For all other (non-in vivo) experiments, samples were allocated to groups randomly.
Blinding	Blinding was not possible in this study for feasibility reasons.

Reporting for specific materials, systems and methods

We require information from authors about some types of materials, experimental systems and methods used in many studies. Here, indicate whether each material, system or method listed is relevant to your study. If you are not sure if a list item applies to your research, read the appropriate section before selecting a response.

Materials & experimental systems

n/a	Involved in the study	
☐	☒	Antibodies
☐	☒	Eukaryotic cell lines
☒	☐	Palaeontology and archaeology
☐	☒	Animals and other organisms
☒	☐	Clinical data
☒	☐	Dual use research of concern
☒	☐	Plants

Methods

n/a	Involved in the study	
☒	☐	ChIP-seq
☐	☒	Flow cytometry
☒	☐	MRI-based neuroimaging

Antibodies

Antibodies used

1) Rabbit monoclonal primary antibody against TRP1 (Abcam, ab178676, Clone: EPR13063)
2) Invitrogen Rabbit IgG isotype control, cat# 10500C
3) TYRP1 antibody (TA-99 clone, BioXCell)
4) Flanvotumab antibody (Mybiosource, 20D7S clone, cat# MBS1563589)
5) Purified Mouse IgG2a, K Isotype Ctrl Antibody (Biolegend, #401502)
6) FITC Anti-human CD45 Antibody (Biolegend, HI30, #304005)
7) BD Pharmingen PE Mouse anti-human CD45 (BD, HI30, #560975)
8) Brilliant Violet 421 anti-human CD4 Antibody (Biolegend, OKT4 #317433)
9) Brilliant Violet 605 anti-human CD8a (Biolegend, RPA-T8, #301039)
10) APC anti-human CD137 (Biolegend, 4B4-1, #309809)
11) PE/Cyanine7 anti-human CD134 (Biolegend, BerACT35, #350012)
12) Alexa Fluor 488 AffiniPure Goat Anti-Human IgG, F(ab')2 fragment specific (Jackson Immunoresearch, Polyclonal, #109-545-006)
13) TYRP1 antibody (Biorbyt, Polyclonal, #orb402977)
14) CD3 Monoclonal Antibody eFLuor450 (ThermoFisher, OKT3, #48-0037-42)
15) CD14 antibody, anti-human, PE (Miltenyi Biotec, TUK4, #130-113-147)
16) CD25 antibody, anti-human, APC (Miltenyi Biotec, 4E3, #130-113-280)
17) CD62L antibody, anti-human, FITC (Miltenyi Biotec, 145/15, #130-113-619)

Unless otherwise stated the concentration of antibody used is the one recommended by the manufacturer's.
Lot number information was not recorded.

Validation

Antibodies were not validated. We used trusted commercial sources and used antibodies following the manufacturer's recommended protocols and applications.

1) Validated by Abcam and references provided in website https://www.abcam.com/products/primary-antibodies/trp1-antibody-epr13063-ab178676.html
2) Validated by Thermo and references provided in website https://www.thermofisher.com/antibody/product/Rabbit-IgG-Isotype-Control/10500C
3) Validated by BioXcell and references provided in website https://bioxcell.com/invivomab-anti-mouse-human-tyrp1-trp1-gp75
4) This antibody has been widely used to target TYRP1, including in the clinical setting (Khalil et al. 2016). Additional references provided in website https://www.mybiosource.com/monoclonal-human-antibody/flanvotumab/1563589
5) Validated by BioLegend and references provided in website https://www.biolegend.com/en-gb/products/purified-mouse-igg2a-kappa-isotype-ctrl-2622
6) Validated by BioLegend and references provided in website https://www.biolegend.com/fr-fr/products/fitc-anti-human-cd45-antibody-707
7) Validated by BD and references provided in website https://www.bdbiosciences.com/en-us/products/reagents/flow-cytometry-reagents/research-reagents/single-color-antibodies-ruo/pe-mouse-anti-human-cd45.560975
8) Validated by Biolegend and references provided in website https://www.biolegend.com/en-us/products/brilliant-violet-421-anti-human-cd4-antibody-7775?GroupID=BLG5901
9) Validated by Biolegend and references provided in website https://www.biolegend.com/en-us/products/brilliant-violet-605-anti-human-cd8a-antibody-7651?GroupID=BLG5903
10) Validated by Biolegend and references provided in website https://www.biolegend.com/nl-be/products/apc-anti-human-cd137-4-1bb-antibody-3910
11) Validated by Biolegend and references provided in website https://www.biolegend.com/en-us/products/pe-cyanine7-anti-human-cd134-ox40-antibody-7234?GroupID=BLG9043
12) Validated by Jackson Immunoresearch and references provided in website https://www.jacksonimmuno.com/catalog/products/109-545-006
13) Validated by Jackson Biorbyt and references provided in website https://www.biorbyt.com/tyrp1-antibody-orb402977.html
14) Validated by Jackson ThermoFisher and references provided in website https://www.thermofisher.com/antibody/product/CD3-Antibody-clone-OKT3-Monoclonal/48-0037-42
15) Validated by Miltenyi Biotec and references provided in the website https://www.miltenyibiotec.com/US-en/products/cd14-antibody-anti-human-tuk4.html#conjugate=viogreen:size=100-tests-in-200-ul
16) Validated by Miltenyi Biotec and references provided in the website https://www.miltenyibiotec.com/US-en/products/cd25-antibody-anti-human-4e3.html#conjugate=vio-bright-fitc:size=100-tests-in-200-ul
17) No validation specified by manufacturer. References for this antibody include Rethacker et al. 2021.

Eukaryotic cell lines

Policy information about cell lines and Sex and Gender in Research

Cell line source(s)	A549, B16-F10 and HEK-293T - ATCC<br>M202 - Male<br>M229 - Male<br>M207 - Female<br>M230 - Male<br>M249 - Female<br>M285 - Female<br>All cutaneous melanoma cell lines were established from patient biopsies under UCLA IRB approval 11-003254.<br>Undifferentiated pleomorphic sarcoma cell lines UPS-03 and UPS-04 were a kind gift from Dr. Anusha Kalbasi (University of California, Los Angeles). Uveal melanoma cell lines MP38, MP41, MP46, MP65, and MM28 were a kind gift from Dr. Chandrani Chattopadhyay (MD Anderson). Acral melanoma cell lines SK-Mel709B, SK-Mel990A, SK-Mel1094A, and SK-Mel1107A, were a kind gift from Dr. Taha Merghoub (Memorial Sloan Kettering Cancer Center, MSKCC).
Authentication	Cell lines were periodically authenticated by STR profiling using GenePrint 10 System (Promega) and were matched with the earliest passage cell lines.
Mycoplasma contamination	Melanoma cell lines were subjected to Mycoplasma tests periodically with the MycoAlert Mycoplasma Detection Kit (Lonza) and yielded negative results.
Commonly misidentified lines<br>(See ICLAC register)	No commonly misidentified cell lines were used.

Animals and other research organisms

Policy information about studies involving animals; ARRIVE guidelines recommended for reporting animal research, and Sex and Gender in Research

Laboratory animals	- Mouse C57Bl/6N - age 6-8 weeks<br>- Mouse NOD/SCID/IL-2Rgnull (NSG) - age 6-8 weeks
Wild animals	No wild animals used
Reporting on sex	All studies were performed in female mice unless otherwise specified. For long-term toxicity studies, both male and female mice were used to examine sex-specific toxicities.
Field-collected samples	No field-collected samples used
Ethics oversight	All animal experiments were performed under the UCLA Animal Research Committee protocols # ARC-2004-159 and ARC-2021-040, which were previously approved by the Institutional Animal Care and Use Committee.

Note that full information on the approval of the study protocol must also be provided in the manuscript.

Flow Cytometry

Plots

Confirm that:

- [x] The axis labels state the marker and fluorochrome used (e.g. CD4-FITC).
- [x] The axis scales are clearly visible. Include numbers along axes only for bottom left plot of group (a 'group' is an analysis of identical markers).
- [x] All plots are contour plots with outliers or pseudocolor plots.
- [x] A numerical value for number of cells or percentage (with statistics) is provided.

Methodology

Sample preparation	TYRP1 intracellular and surface protein expression was detected by flow cytometry. Briefly, tumor cells were seeded and grew for 24-48h. For internalized staining, cells were treated with 2.5μg of TYRP1 antibody and monensin (BD GolgiStop) to block internalized protein degradation for 16h in culture, following the manufacturer's recommendations. Cells were detached from the culture plate using 2mM EDTA (ThermoFisher Scientific) and transferred to V-bottom plates to perform the intracellular and surface stain. Cells were first stained with Zombie Violet (Biolegend) 1:100 dilution for 15min at room temperature, and 50uL of FBS was added to block the unspecific staining. For surface staining, 2.5μg of TYRP1 antibody or Mouse IgG2a, k Isotype Control were added and incubated for 30min on ice. After washing, Anti Mouse IgG2A, k-APC was used as a secondary antibody. For intracellular and internalized staining, the cells were permeabilized using Fixation and Permeabilization kit (BD biosciences) according to manufacturers' instructions and stained with the same primary and secondary antibodies as used for surface stain.

	To measure T-cell activation or transduction, T cells were collected, washed with PBS, and stained with Zombie Violet live/dead stain (1:100, Biolegend) in PBS. After incubation, cells were stained with corresponding antibodies for 30min on ice. In all flow cytometry staining experiments, after the last incubation with antibodies, cells were then washed, fixed, and stored at 4°C until flow cytometry acquisition. All stains and washes were performed in PBS unless otherwise indicated. For in vivo flow cytometry analysis, tumors were digested with 50u/ml DNase (Sigma) and 1mg/ml Collagenase (Sigma) for 1h at 37C. Spleens were dissected manually. Single-cell suspensions were obtained using 70um strainers. 1x106 cells were stained with Zombie NIR (Biolegend) 1:100 for 15min at room temperature. After a wash, cells were blocked with anti-mouse CD16/32 (Invitrogen) and stained with anti-mouse CD3-eFluor450 (eBiosciences) or anti-human CD45-FITC (Biolegend) for 30min at 4C.
Instrument	Attune NxT (Invitrogen) and LSRII (BD)
Software	FlowJo v10
Cell population abundance	NA
Gating strategy	Gating strategies are depicted in Supplemental figures 1 and 2. Unstained samples were used to determine 'positive' populations.

☒ Tick this box to confirm that a figure exemplifying the gating strategy is provided in the Supplementary Information.
